# Paraparesis and Disseminated Osteolytic Lesions Revealing Cholangiocarcinoma: A Case Report

**DOI:** 10.25122/jml-2020-0068

**Published:** 2020

**Authors:** Silvina Ilut, Vitalie Vacaras, Paula Rosu, Aurora Muntiu, Constantin Dina

**Affiliations:** 1. Department of Clinical Neurosciences, “Iuliu Hatieganu” University of Medicine and Pharmacy, Cluj-Napoca, Romania; 2. Neurology II Department, County Emergency Hospital, Cluj-Napoca, Romania; 3. Department of Radiology “Ovidius” University, Faculty of Medicine, Constanta, Romania

**Keywords:** Cholangiocarcinoma, bone metastasis, paraparesis

## Abstract

Bone metastases in cholangiocarcinoma are uncommon. We report the case of a patient with disseminated osteolytic lesions who was admitted to the Neurology Department for progressive paraparesis. On the computed tomography examination, specific features for cholangiocarcinoma were described, confirmed later by the histopathological aspect of the bone lesions.

## Introduction

Cholangiocarcinoma (CCA) is a malignant tumor, and its origin can be the liver or the extrahepatic bile ducts [1]. Usually, patients with cholangiocarcinoma have locally advanced disease at the moment of diagnosis with distant metastasis being uncommon [2] since patients typically develop distant metastasis in the late stages of the disease. It commonly spreads to the regional lymph nodes, liver, and lungs. However, bone metastases from cholangiocarcinoma are rare compared to other tumors [3]. The present text aimed to report a case of CCA with multiple bone metastases initially thought to be multiple myeloma, CCA being an incidental finding.

## Case Report

**Figure 1:**
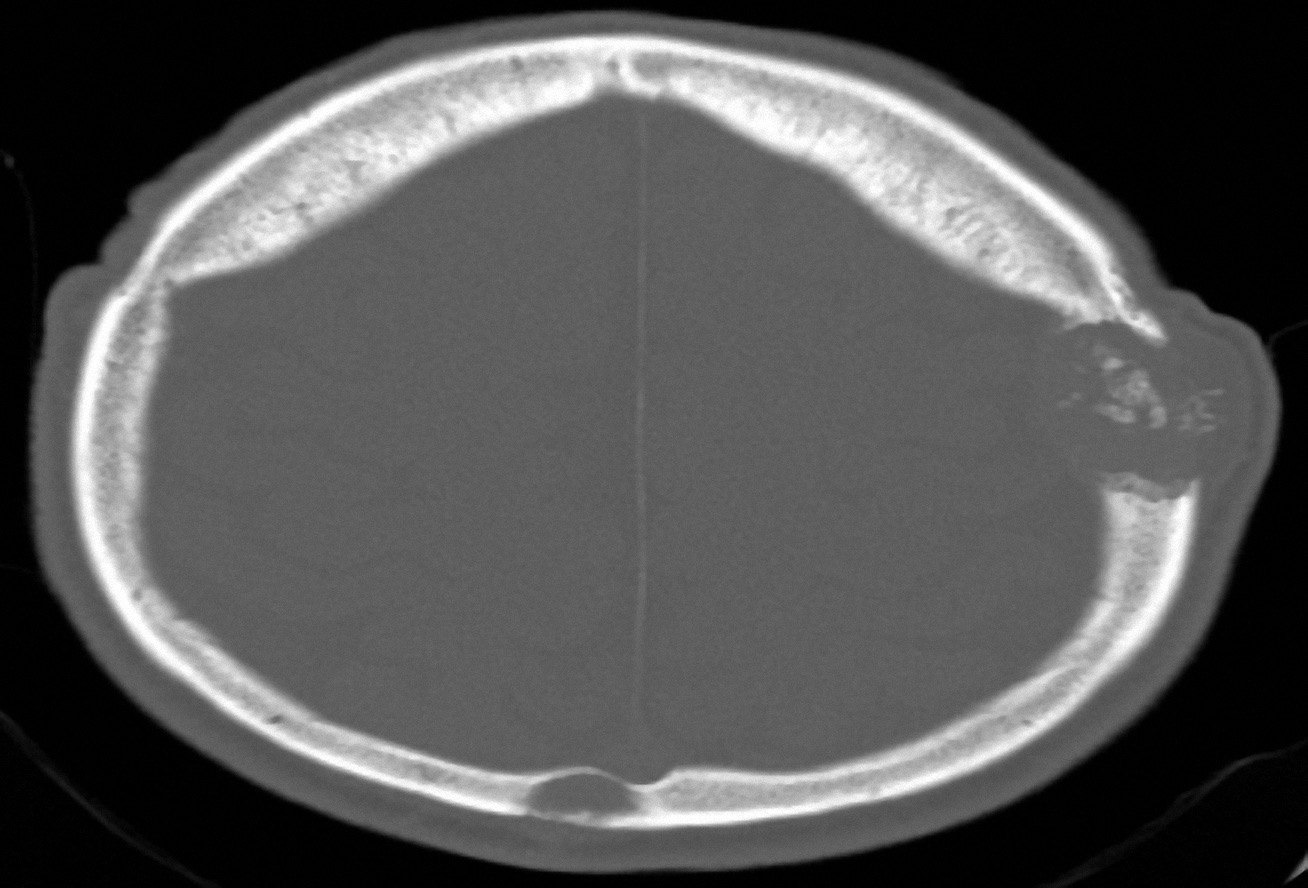
Cerebral CT scan – bone window: osteolytic lesion in the left frontal bone.

A 60-year-old male patient, a previous smoker and alcohol user, was admitted to the Neurology II Clinic exhibiting progressive paraparesis for over six months, ostealgia, muscular pain, and paresthesias in the lower extremities. The symptoms have had a gradual onset and have increased in the past three weeks before the presentation due to an accidental falling episode. The patient underwent right femoral surgery in 1996, and a non-MRI compatible metallic plate was placed. The pelvic, femoral and lumbar spine radiography performed in the emergency department showed multiple osteolytic lesions. The cerebral CT scan (Figure 1) revealed osteolytic lesions localized in the calvarium and cervical spine.

The first neurosurgical consult recommended a spine MRI exam. However, this was not possible due to the non-MRI compatible right femoral metallic plate. The neurological examination revealed: flaccid paraparesis, and gait was possible within small distances, showing generalized muscular atrophy.

An extensive blood panel was performed: PSA, alpha-fetoprotein, carcinoembryonic antigen and carbohydrate antigen 19-9 which had normal values, microscopic blood sample with normal characteristics, alkaline phosphatase and gamma-glutamyl transferase – increased values 169 U/L respectively 126 U/L, serum protein electrophoresis – slightly increased alfa 2 globulins (a value of 12.13%), LDH was slightly increased: 254 U/L, mild hyposideremia. The urine analysis showed increased 24-hour proteinuria - 226,5 mg/24h, but IgA, M and G had normal values. An electroneuromyography examination was performed, and it revealed sensitive axonal polyneuropathy. Due to the radiological characteristics of the osteolytic lesions, the high suspicion of multiple myeloma was raised, and therefore, the patient was referred to the hematology department. The diagnosis of multiple myeloma or POEMS (Polyneuropathy, Organomegaly, Endocrinopathy, Monoclonal protein, Skin changes) syndrome was excluded (the medullary biopsy was normal).

**Figure 2:**
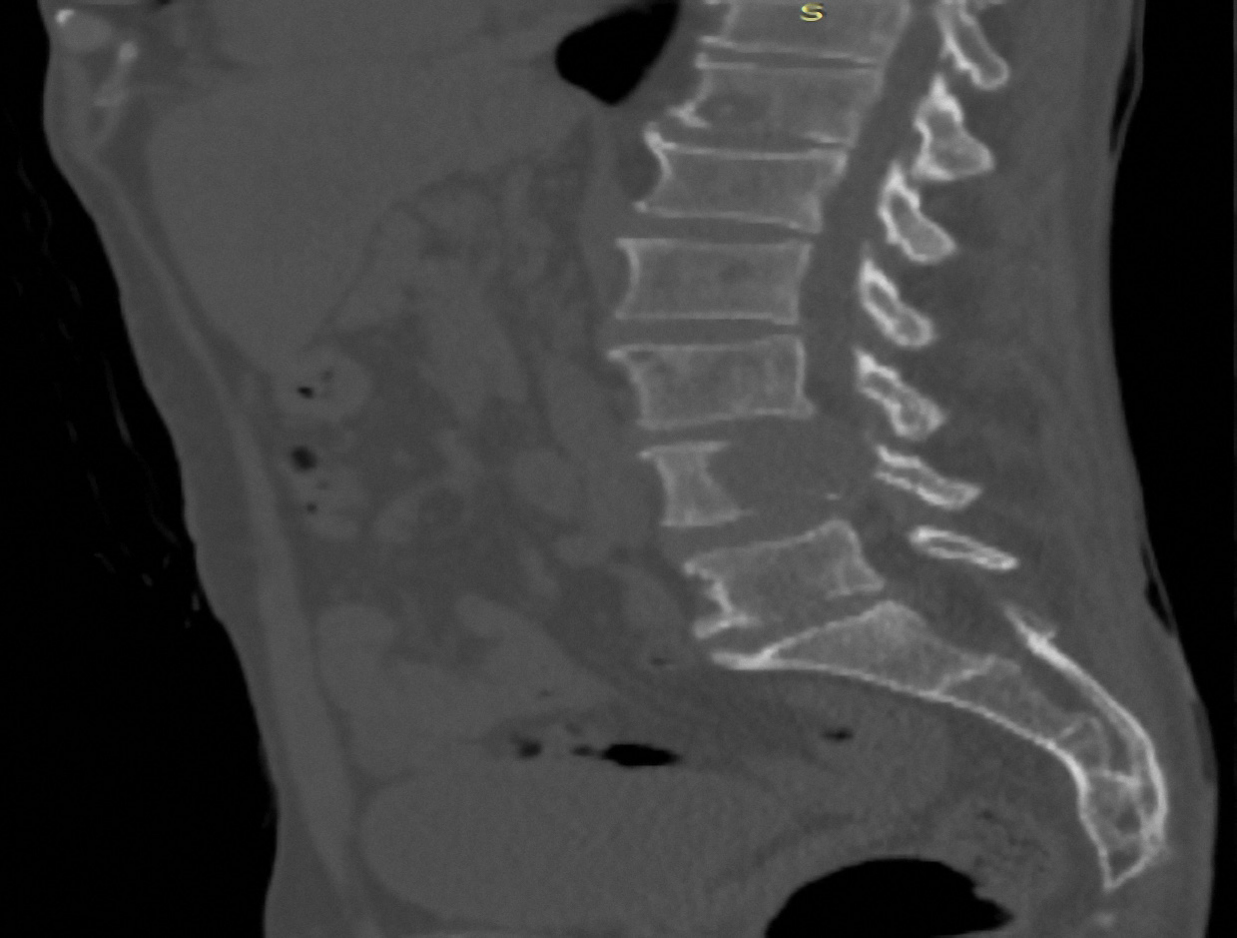
Thoracic-abdominal-pelvic (TAP) CT scan – bone window: osteolytic lesion in the L4 vertebral body with intracanalar extension and narrowing of a third of the vertebral canal.

**Figure 3:**
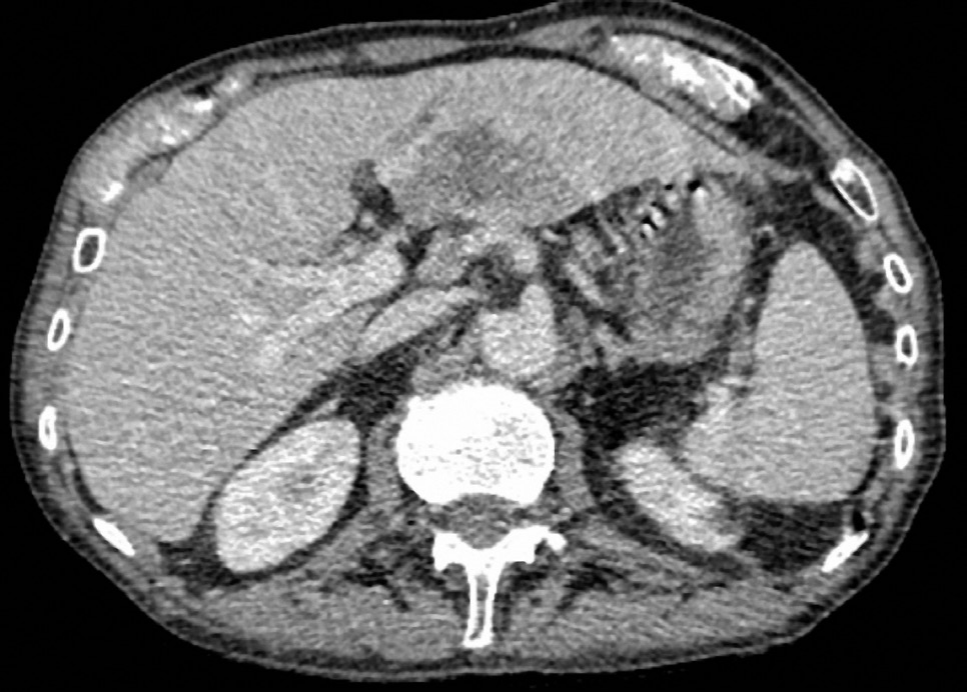
TAP CT scan - venous phase: hypodense elongated structures in the left hepatic lobe; adjacent to this area, there is a round-oval lesion (16 mm).

The contrast-enhanced thoracic-abdominal-pelvic CT scan showed multiple osteolytic lesions: in the right iliopubic bone, right and left iliac bone, left femoral bone, the L4 vertebrae (Figure 2) and the D3 and D8 vertebrae with extension towards the vertebral canal and in close contact with the spinal cord. The CT scan also showed hypodense elongated structures in the left hepatic lobe. Adjacent to this area, there was a round-oval lesion measuring approximately 16 mm and with fluid density. Post-contrast, in the arterial phase, the left hepatic lobe was more opacified. The venous phase (Figure 3) showed a reduced caliber of the portal vein and weak opacification. The hepatic lesion raised a high suspicion of cholangiocarcinoma.

The patient underwent left frontoparietal craniectomy, and a week later, he suffered D7 and L4 vertebrae decompressive hemilaminectomy and removal of the D4 and L4 vertebral osteolytic lesions. However, a few days later, during passive movement, he suffered a left proximal femoral fracture. Closed reduction and osteosynthesis were performed. Postoperatively, he was transferred to our clinic. Upon neurological reevaluation, there was a substantial decrease in the pain level.

**Figure 4:**
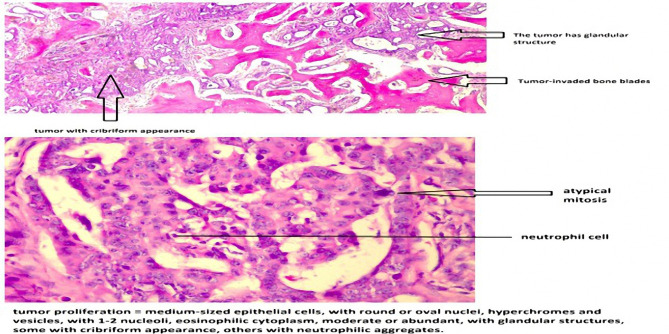
The histopathological aspect of the bone samples.

**Figure 5:**
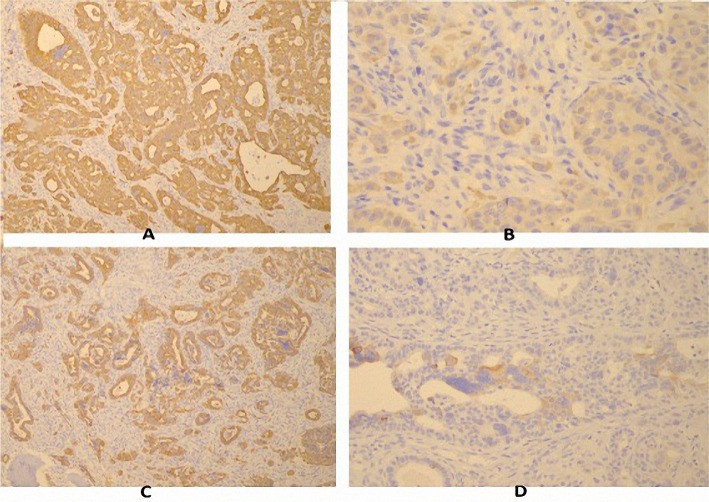
Immunohistochemistry from bone samples: A: CK7 positive (100x); B: CK8 positive (100x); C: CK19 positive (400x); D: CK 20 positive (100x). The patient was referred to an oncologist, but currently, there are no curative treatment options available, since the patient is in the terminal phase of the disease.

The histopathological examination of the bone samples revealed the following aspects: macroscopically, several yellow-gray fragments were examined and focally rigid–bone areas, some friable, were noticed; microscopically, various bone trabeculae and cartilage, diffusely infiltrated by a tumoral proliferation composed of small and medium tumoral cells with round or oval, hyperchromatic or vesicular nuclei, with marked desmoplasia, reduced inflammatory infiltrate and decreased areas of necrosis and hemorrhage (Figure 4) were seen. Immunohistochemistry analysis showed diffuse intense CK7 positivity, focally intense CK20 positivity, diffuse CK19 positivity, diffuse EMA positivity, isolated, low positivity for CA 19.9, and negative TTF-1 (Figure 5). The histopathological exam concluded that the previously described features belong to adenocarcinoma metastasis, possibly with a biliopancreatic origin. The results correlated with the clinical context came to confirm the radiological suspicion of bone metastasis from cholangiocarcinoma.

## Discussion

Cholangiocarcinomas (CCAs) are malignancies of the biliary duct system [4]. Biliary duct system malignancies are less than one percent of all human types of cancer and approximately 10% to 15% of all primary liver malignancies, occurring mostly in the seventh decade of life, with a male predominance - male: female ratio of 1.2–1.5:1.0 [5].

Several risk factors for cholangiocarcinoma were identified: cholestatic liver diseases, liver cirrhosis, biliary stone disease, diseases caused by toxins - alcohol, tobacco, chemical toxins, metabolic conditions [6]. In this case, the patient had no significant clinical history except smoking and alcohol consumption.

Generally, the evolution of the disease is prolonged; patients may experience weight loss, jaundice, abdominal pain or discomfort, and sometimes night sweats [7]. In this case, the patient presented weight loss, motor impairment of the lower limbs, and diffuse bone pain, which are complications of the distant extension of the disease.

Few cases of bone metastasis from CCA are described in the literature, and the most commonly reported sites were the humerus, the fibula, the femoral bone [8], and the scapula [9-11]. In our case, multiple osteolytic lesions with a malignant aspect were discovered in the coxal bone, the sacrum, and the left proximal femoral region, as well as in the calvarium and spine.

Considering that the path to diagnosis started from secondary injuries, the difficulty of this case was to find the source. Osteolytic lesions occur most often in multiple myeloma, melanoma, renal cell carcinoma, non-small cell lung cancer, thyroid cancer, Non-Hodgkin lymphoma, thyroid cancer, in Langerhans-cell histiocytosis and rarely in cholangiocarcinoma [12,13]. In this case, the limitation is the impossibility of carrying out the investigation, caused by the fact that the patient had a history of right femoral surgery with a metallic plate which was not compatible with MRI.

Another tool to help diagnose CCA are tumor markers, but in this case, the values of these markers were within the normal range, despite the advanced stage of the disease. Taking into account that the patient was in an advanced stage of the disease, it is essential to have a histological or cytological confirmation, which can be obtained through endoscopic ultrasound or percutaneous biopsy, ultrasound-guided, depending on the location of the lesion [5]. For this patient, the method of choice would have been percutaneous biopsy.

The CCA median survival is about 12 months. The prognosis is unfavorable in CCA because of the lack of response to chemotherapy and radiotherapy [14, 15]. The only option that remains is palliative care, symptomatic therapy – pain management – in order to improve the patients’ quality of life.

Therefore, the approach, in this case, should be multidisciplinary and should include an oncologist, gastroenterologist, psychologist, radiotherapist, neurologist, neurosurgeon, and orthopedic surgeon.

## Conclusion

Cholangiocarcinoma with bone metastases is a rare condition with an unfavorable prognosis. Unfortunately, most patients present in advanced stages when the tumor is unresectable because of the silent evolution and the nonspecific symptomatology. This case of advanced biliary tract cancer with neurological symptomatology – pain and paraparesis- highlights the unusual site of metastases for cholangiocarcinoma. It also shows the difficulty of obtaining a diagnosis when facing nonspecific symptomatology and also marks the importance of a multidisciplinary approach.

## Conflict of Interest

The authors declare that there is no conflict of interest.

## Acknowledgment

We want to thank the Histopathology Department of the Cluj-Napoca County Emergency Hospital for their involvement.
